# Purification and use of crude green glycerol from the transesterification of triglycerides in the formulation of an alcohol gel hand sanitizer

**DOI:** 10.1038/s41598-023-49422-5

**Published:** 2024-03-06

**Authors:** Tassio Trindade Mazala, Mateus Costa Viana, Guilherme Carneiro, David Lee Nelson, Maria B. de Freitas-Marques, Bruno Spinosa De Martinis, Jacques Florêncio, Fernanda Marur Mazzé, Severino G. Domingos da Silva, Sandro L. Barbosa

**Affiliations:** 1 Department of Pharmacy, Universidade Federal dos Vales do Jequitinhonha e Mucuri-UFVJM, Campus JK, Rodovia MGT 367 - Km 583, n° 5.000, Alto da Jacuba, Diamantina, Minas Gerais 39100-000 Brazil; 2 Department of Chemistry, Instituto de Ciências Exatas, Universidade Federal de Minas Gerais, Av. Antônio Carlos, 6627, Pampulha, Belo Horizonte, Minas Gerais 31270-901 Brazil; 3 Universidade de São Paulo, Faculdade de Filosofia, Ciências e Letras de Ribeirão Preto, Av. Bandeirantes, 3900, Ribeirão Prêto, SP 14040-900 Brazil; 4 Curso de Farmácia. Faculdade de Minas, Faminas-BH, Av. Cristiano Machado, 12001, Vila Clóris, Belo Horizonte, Minas Gerais 31744-007 Brazil; 5 Institute of Chemistry, Federal University of Rio Grande do Norte, Natal, RN 59078-900 Brazil

**Keywords:** Biochemistry, Biological techniques, Biophysics, Biotechnology, Chemical biology, Chemistry

## Abstract

The aim of this study was to produce an alcohol gel hand sanitizer containing green glycerol. Crude glycerol was purified using chemical and physical treatments. The sanitizer was prepared using 71.100 g of 99.3° GL ethanol, 28.0 g H_2_O, 0.5 g of Carboxypolymethylene [Carbopol 940® or Carbomer], 5 drops of triethanolamine (pH 5–7), and glycerol (1.5% w/w). The thermal behavior of the ethanol, carbopol, triethanolamine, glycerol, and alcohol gels were evaluated using Thermogravimetry and Differential Thermal Analysis. The apparent viscosity was obtained using a rotary viscometer. The determination of in vitro spreadability was achieved by an adaptation of the Knorst method. The ethanol content was measured by headspace gas chromatography using a flame ionization detector. The thermal behavior of the gels was influenced by the presence of glycerol, which confirms the possible network interactions formed. The relative densities of the samples were between 0.887 and 0.890 g/cm^3^. No alteration of the pH of the formulation resulted from the incorporation of glycerol. The apparent viscosities of the alcohol gels were greater than 20,000 cP. No alteration in the in vitro spreadability of the gel alcohol (530.6 mm^2^) resulted from the addition of glycerol. Hand sanitizer was produced using glycerol from a transesterification reaction. It represents an alternative use for the glycerol being produced in biodiesel processes. The product satisfied the requirements of WHO that preconize a formulation containing 1.45% glycerol as an humectant to protect skin against dryness and dermatitis.

## Introduction

Glycerol, also called glycerine or glycerin, is a simple three-carbon triol. It is a colorless, odorless, viscous liquid that is sweet-tasting and non-toxic. It is the principal by-product of the production of fatty acid methyl esters (FAME or biodiesel). Every processing cycle yields approximately 10% (w/w) of crude glycerol, and, therefore, it is responsible for a glut in the glycerol market. The underutilized byproduct has undoubtedly increased the environmental liability. There are many studies focusing on discovering effective applications to produce chemical commodities of greater value to ensure sustainable consumption and production patterns^1^. The properties of glycerol and its use in formulations of polymers and in various other applications are mentioned by Ben et al.^1^

Glycerol serves as a lubricant in the textile industry^2^, as well as in equipment and materials that come in contact with food, pharmaceuticals, cosmetics or skin^3^. In special cases, it is also employed as a hydraulic fluid^4^. It is used as a solvent, humectant, plasticizer, and sweetener^5^; in the production of nitroglycerine (dynamite)^6^, liquid soap^7^, liquors^8^, confectionery^9^, blacking^10^, printing and copying inks^11^, elastic glues^12^, lead oxide cements^13^; to keep fabrics pliable^14^, to preserve printing on cotton^2,15^, for printing rollers^16^, hectographs^17^; to keep frost off windshields^18^; as an antifreeze in automobiles^19^, gas meters and hydraulic jacks^20^, and in shock absorber fluids^21^. It is used as a nutrient in fermentation fluids^22–24^. Glycerol is also used in home-brewed spirits to give them a smoother or mellower flavor^25^, and it is employed as a precursor for the synthesis of various other products of greater value^26,27^.

Glycerin serves as a moisturizing substance (humectant), which reduces skin dryness and prevents household chemicals from drying out. This fact is extremely important because rapid water loss is an unfavorable phenomenon because it makes application difficult. Drying of such preparations may also lead to changes in their appearance, such as cracking of the surface, loss of product weight^28^, etc. Glycerin improves the functional properties of the preparations by preventing crystallization of the ingredients, influencing rheological properties, providing appropriate smoothness and uniformity, and influencing proper distribution and adhesion. The use of glycerin as a component in preparations for cleaning hard surfaces is beneficial because it limits the drying of household chemicals, reduces the drying of the skin during use and the cost is relatively low because it is a secondary product of biodiesel production. Applications for glycerol are important because its accumulation is detrimental to the environment^29^.

Emilia Klimaszewska^30^, studied the possibility of using glycerol in cleaning preparations for hard surfaces, innovative raw materials, determining the high quality of the final product and the selection of quality characteristics of cleaning products. Based on literature reports, market analysis, and the author’s experience, the selection of raw materials for three types of cleaning preparations: pastes, milks and powders was made.

Glycerol helps to increase the viscosity of liquid drug formulations, pharmaceutical syrups, etc.^31^. It also acts as a humectant for medicinal pills or tablets. It is used in cement compounds^32^, caulking compounds, and pressure media. It is also used in embalming fluids, masking and shielding compounds, soldering compounds, and compasses; wetting agents, emulsifiers, skin protective products, asphalt, ceramics, photographic products, leather, wood treatments, and adhesives^33^.

The ethanol-based handrub (EBHR) formulation of the World Health Organization (WHO) contains 1.45% glycerol as a humectant to protect healthcare workers’ (HCWs) skin against dryness and dermatitis^34^. However, glycerol seems to negatively affect the antimicrobial efficacy of alcohols. In addition, the minimal concentration of glycerol required to protect hands remains unknown.

Menegueti et al.^34^ studied formulations containing various concentrations of glycerol, and they determined that a formulation containing 0.5% glycerol was more beneficial for the person’s skin. We produced a gel containing 1.5% (w/w) glycerol based on the WHO EBHR formulation for use by healthcare workers in a tropical climate setting.

Our research group recently used activated charcoal prepared from the endocarp of *Acrocomia aculeata* (macaúba) by ZnCl_2_ activation for the adsorptive purification of pretreated crude glycerol (CG)^35^. In another study, we produced glycerol by transesterification of triglycerides from waste cooking oil using red mud (RM) composed of a waste alkaline solution (pH = 13.3) obtained from the production of alumina as a catalyst^36^. In the present study, the purified glycerol obtained from transesterification of recovered cooking oil was employed in the formulation of an alcohol gel hand sanitizer.

## Experimental

### Raw materials and chemicals

All the reagents were HPLC grade. Ethanol (99.5% purity), acetonitrile (ACN, 99.8%), acetone (99.8%), ethyl acetate (99.0%), and triethanolamine (99.0%) were obtained from Sigma-Aldrich (St. Louis, MO, USA). Acetaldehyde (99.0%) methanol (99.9%), and butanol (99.0%) were purchased from Merck (Darmstald, Germany). Isoamyl alcohol (98.5%) and isobutyl alcohol ([ISO]; 99.0%) were obtained from Labsynth (Diadema, Brazil). Benzene (99.0%) and toluene (99.5%) were purchased from Reagen (Colombo, Brazil). Deionized water was obtained using a Milli-DI water purification system from Merck. Ultrapure water was used in the blank gel matrix^37^. [Carbopol 940] was obtained from Vetec (São Paulo, Brazil).

### Purification of the crude glycerol

Purification of crude glycerol was performed using a combination of chemical and physical treatments with solvent extraction, as described in the literature^35^. A 200-mL portion of crude glycerol was obtained from the transesterification of waste cooking oil with methanol using KOH as the catalyst^38^. The methanol from the transesterification process was evaporated with the aid of a rotary evaporator, and the pH of the crude glycerol residue (135.10 g) was adjusted to pH 2 with H_3_PO_4_ (85%), followed by shaking for 1 h at a constant rate of 200 rpm and allowing the mixture to stand for 12 h for phase separation by decantation.

The top layer contained FAME, fatty acids, and soap; the middle layer was rich in glycerol; and the bottom layer contained a salt-rich solid (K_2_HPO_4,_), which was filtered. The liquid mixture was transferred to a separation funnel, the upper phase was separated, and the intermediate glycerol phase was neutralized by the addition of KOH pellets to pH 7.0, centrifuged to remove the salt and extracted with propanol; the solvent was separated from glycerol using a rotary evaporator. All the experiments were performed in triplicate. The adsorptive purification of the pretreated crude glycerol was achieved at room temperature (25 °C) and ambient pressure using a column (15.0 cm height by 1.0 cm diameter) containing activated charcoal (10 g)^35^. Small aliquots of crude, pretreated glycerol was added to the column until a total volume of 1 L of glycerol was added. The adsorption lasted 48 h. The purity of the filtered glycerol was determined by ^1^H-NMR according to the procedure described in Barbosa et al.^35^. ^1^H– and ^13^C-NMR spectra were recorded on a Bruker Avance 400 Spectrometer. The regeneration of used activated charcoal was performed after shaking at 250 rpm with methanol in the ratio of 3:1 (v/w) for 1 h to remove the adsorbed glycerol.

The activated charcoal was separated from the solution by filtration, rinsed with hexane to remove the excess solvent, and heated in a muffle furnace at 150 °C for 5 h for reactivation. After using the charcoal three times, it no longer retained all of the pigments and required reactivation by heating with ZnCl_2_.

### Preparation of the alcohol gel hand sanitizer

To a beaker (500 mL) containing 71.100 g of 99.3° GL ethanol was added 28.0 g of distilled water, with magnetic stirring for approximately 10 min, and 0.5 g of the anionic acrylic acid polymer (Carbopol 940^→^) was dispersed in the hydroalcoholic solution. After complete dissolution, five drops of triethanolamine (pH 5–7) were slowly added. This alcohol gel hand sanitizer was coded HS1. Glycerol (1.5% w/w) was added to the final product in accordance with the recommendation of the WHO^34^ (coded HS2). The gels were stored in transparent polypropylene bottles with a capacity of 100 g.

### pH determination

The samples were diluted in previously neutralized water (pH 7.0) at a concentration of 10% (w/v), and pH measurements were directly conducted in triplicate using a model mPA210 pH meter (MS Tecnopon Instrumentation, Porto Alegre, Brazil)^39^.

### Relative density

The relative density was determined using a clean and dry 25-mL pycnometer at a temperature of 25 °C. The specific gravity was calculated as the ratio of the net weight of the samples to the net weight of water^40^. The densities were measured in triplicate.

### Thermal behavior

The thermal behavior of the ethanol, carbopol, triethanolamine, glycerol, and alcohol gels were evaluated using simultaneous Thermogravimetry (TG) and Differential Thermal Analysis (DTA). The TG curves were derived in 1st order (DTG) to confirm the thermal phenomena. The simultaneous TG/DTA curves were obtained on the DTG60H Shimadzu thermobalance in a dynamic nitrogen atmosphere at 50 mL min^−1^, with a heating rate of 10° min^−1^ from 30 to 300 °C. An open alumina crucible and a sample mass of exactly 3.0 mg were used. The mass loss during heating is expressed as “Δm”.

### Apparent viscosity

The apparent viscosity was obtained using the microprocessed QUIMIS Q860M21 Spindle 4 (rotating rod) rotary viscometer; the speed of 20 rpm was selected in accordance with the regulatory standards^41^, which addresses the quality control of alcohol gels. The spindle was vertically immersed in the sample at room temperature (25 °C) to ensure the absence of bubbles near the spindle, and the viscosity was directly determined in triplicate^38,42^.

### In vitro spreadability

The determination of in vitro spreadability was performed according to an adaptation of a method previously described by Knorst^43^. A circular glass mold plate with a diameter of 20 cm and a thickness of 0.2 mm, equipped with a central hole of 1.2 cm in diameter, was placed on top of a square glass support plate (20 cm × 20 cm). A sheet of graph paper was positioned beneath these plates. The sample was introduced into the hole in the plate and the surface was leveled with a spatula. The mold plate was carefully removed, and an acrylic plate with a predetermined weight (4.7 g) was placed on top of the sample. After one minute, the covered surface area was calculated by measuring the diameter in two perpendicular directions, followed by the calculation of the average diameter.

Spreadability (S), determined at 25 °C, was calculated using the following equation:

$$S = d^{2}.\pi /4$$

where S = spreadability of the sample for a weight of 4.7 g (mm^2^), and d = average diameter (mm).

### Ethanol concentration

The sample (0.10 g) was transferred to a 20 mL HS vial; 15 mL of deionized water and 20 mL of internal standard (ISO) for quantitative analysis were added. The samples were stirred on a vortex stirrer for 10 s, incubated at 90 °C for 6 min, and 400 µL of the vapor phase was injected into a Agilent 7890A, Agilent Technologies Inc., Santa Clara, CA, USA (GC/FID system) using an Agilent GC autosampler 80 (Agilent Technologies Inc.). The separation was achieved using a CP-Wax 52 CB capillary column (30 m × 0.25 mm; 0.25 mm). The oven temperature was initially held at 50 °C for 2 min, followed by an increase at 20 °C/min to 200 °C. The total run time was 9.5 min. Nitrogen 5.0 (99.999% pure) was used as the carrier gas at a constant flow rate of 1.0 mL/min. The injector port temperature was 250 °C, and the injections were performed in the split mode (50:1). The FID temperature was 300 °C, with hydrogen and air flow rates of 40 and 400 mL/min, respectively. The Agilent OpenLAB CDS software was used for data acquisition (Agilent Technologies Inc., Santa Clara, CA, USA)^37^.

### Statistical analysis

The results were represented as mean value ± standard deviation from determinations in triplicate. The Student *t*-test was used to analyze the statistical differences between the mean values (α = 0.05).

## Results and discussions

The purity of the crude glycerol by-product from conventional industrial biodiesel plants is of key economic and technological concern. Small biodiesel industries usually discard the glycerol by-product as waste. The crude glycerol contains large amounts of several impurities that inhibit its direct usage or consumption in industries. The quality of crude glycerol depends on the characteristics of the biodiesel process used at the manufacturing facility. Crude glycerol of 50% purity contains methanol, mono-, di-, and triglycerides, methyl esters and other organic matter. It has few direct uses and has a low commercial value. Even its value as a fuel is marginal. Depending on the types of catalytic route and separation process used, the glycerol purity can reach 80–95%. Crude glycerol was produced by transesterification of waste cooking oil^36^ and purified by filtration through activated charcoal obtained from *Acrocomia aculeata*^35^. The method employed using only filtration through activated charcoal after the pretreatment with H_3_PO_4_ to remove the KOH furnished glycerol of 95.99% purity. The remaining 4% consisted of water. The only difference from commercial glycerol regarding purity was the presence of water.

### ^1^H NMR analysis of the purified glycerol

**Figure 1:**
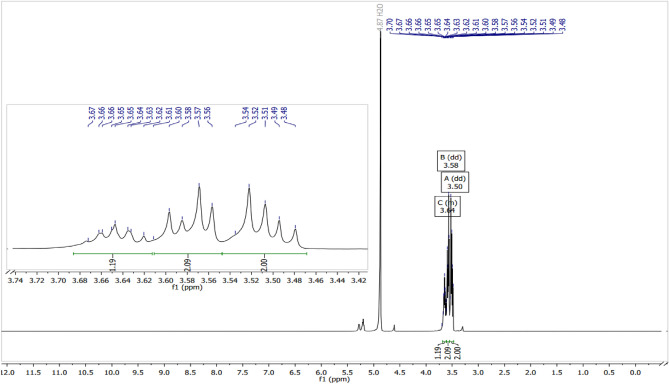
^1^H NMR (400 MHz, CDCl_3_) of purified glycerol, as described in the literature^35^.

The high degree of purity of the glycerol obtained using treated charcoal was confirmed by the ^1^H NMR analyses (Fig. 1). In Fig. 1, a double doublet (J 4 Hz) centered at 3.5 ppm and a double doublet (J 4 Hz) between 3.54 and 3.60 ppm correspond to the methylene hydrogens of glycerol. The multiplet from 3.62 to 3.68 ppm corresponds to the methine hydrogen. The signal for the hydroxyl hydrogens was observed at 4.8 ppm. The data are similar to that described in the literature by Barbosa and collaborators, according to reference^35^.

### Characterization of the gel alcohol hand sanitizer

Both samples (that containing only alcohol and that containing alcohol with glycerol) exhibited a pH close to neutral, approximately 7.5–7.7 (Table 1), with no significant difference between them (*p* > 0.05). Thus, the incorporation of glycerol did not significantly alter the pH of the final formulation^44^. Triethanolamine plays the role of a pH regulator to neutralize the acidic dispersion of the carbomer, thereby elevating the pH level while simultaneously increasing the viscosity and stability of these formulations^45,46^. Thus, the exact amount of triethanolamine used has a direct impact on the pH of the carbomer gel. The neutral pH of the formulation becomes particularly relevant in the development of gel alcohol, as it affects the effectiveness against specific viruses, such as Norovirus, which has been shown to be less resistant to neutral-alcohol-based sanitizers^47^.

**Table 1:** Characterization of gel alcohol in the hand sanitizer with (HS2) and without (HS1) glycerol.

Parameters	HS1	HS2
pH	7.54 ± 0.13	7.69 ± 0.05
Relative density (g/cm^3^)	0.890 ± 0.006	0.887 ± 0.0009
Apparent viscosity (cP)	27,070 ± 62	27,040 ± 35
In Vitro spreadability (mm^2^)	530.6 ± 0.82	530.6 ± 0.82

The relative densities of the products were between 0.887 and 0.890 g/cm^3^, a range commonly found in the literature for gel alcohols^48^. The addition of glycerol did not significantly affect the relative density (*p* > 0.05). The addition of glycerol can affect the hydrogen bonds of the carbomer and influence its viscoelasticity, and it can form a denser elastic structure with the addition of triethanolamine^49^. However, the product density was not significantly altered.

Both alcohol gels exhibited a high average apparent viscosity, greater than 20,000 cP, with no statistically significant difference between them (*p* > 0.05). The viscosities of the products were greater than 8000 cP, as recommended by ANVISA (2002)^41^. The high viscosity observed in the formulation can be attributed to several factors, especially the composition of the formulation. Although the viscosity can be low as a result of the presence of ethanol, the high viscosity of the formulation is directly associated with the concentration of the carbomer, neutralized by triethanolamine in the dispersion^50,51^. Hydrogen bonding interactions between the carbomer and triethanolamine, facilitated by the hydroxyl group of triethanolamine and the carbonyl group of the carbomer, increase the interactions present in the gel formulation, thereby increasing the gel viscosity. With a higher viscosity, enhanced adhesion, reduced dispersibility, and extended drying time can exist^52^.

Finally, the in vitro spreadability test was conducted to assess the ability of a formulation to distribute evenly when applied to the skin. The addition of glycerol did not change the in vitro spreadability of the gel alcohol, which remained at 530.6 mm^2^ for both samples, with no significant difference between them (*p* > 0.05). Indeed, whereas the spreadability can be considered to be relatively low, it is directly correlated with the high viscosity observed in the formulation^53^. Comparatively, gels used for miltefosine delivery exhibited even lower spreadability when determined by the same method, with values around 390 mm^2^^54^.

### Ethanol concentration

The ethanol concentration in all the samples was determined by GC/FID. Two previously prepared products were also analyzed. All the samples contained ethanol concentrations equal to or greater than the concentration considered to be effective (70–80%)^55,56^. A simple and rapid HS-GC/FID method to quantify ethanol in ethanol-based gel hand sanitizers was developed, validated, and applied to the prepared samples.

### Thermal behavior

**Figure 2:**
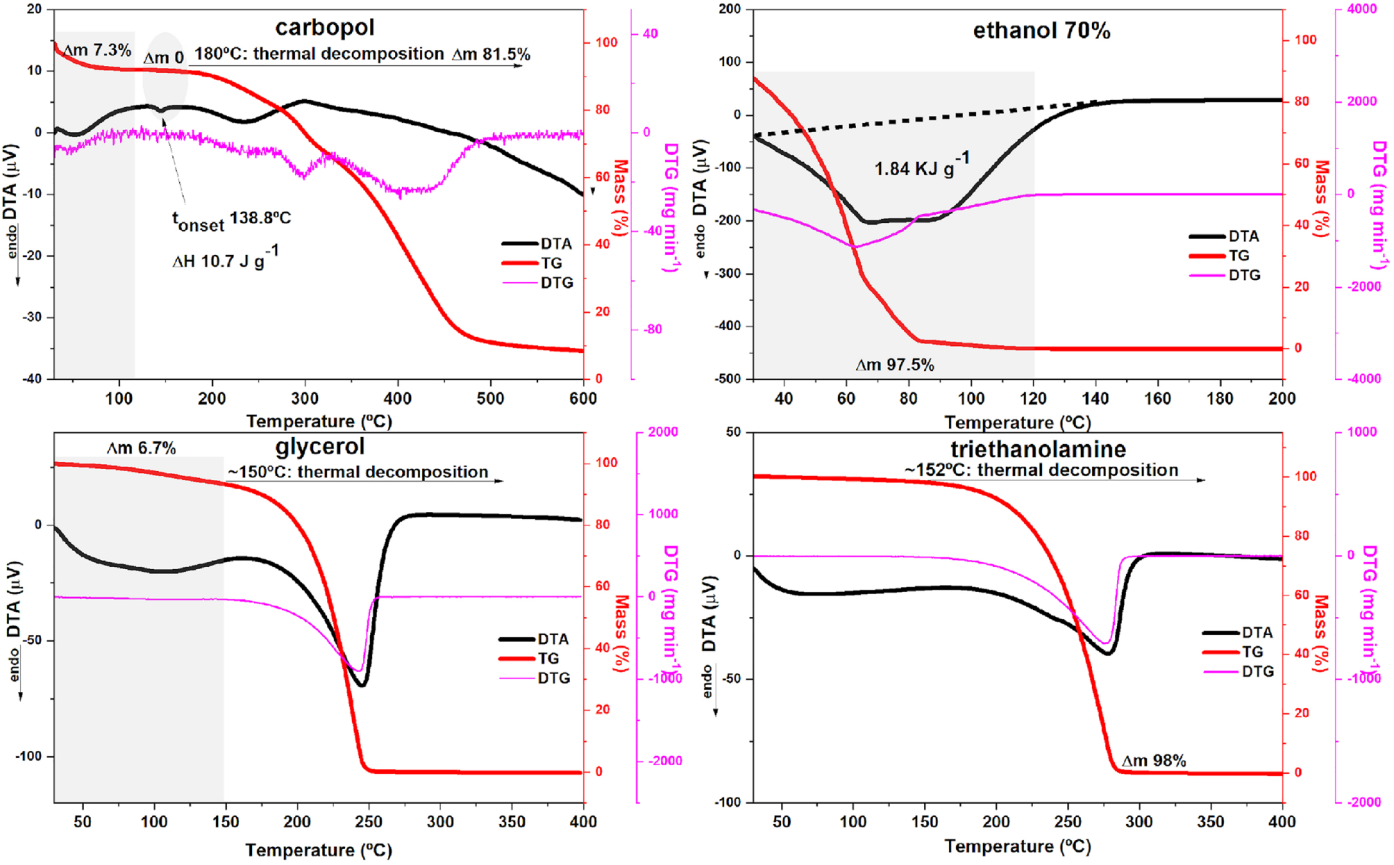
Thermal behavior of Carbopol, 70% ethanol, glycerol and triethanolamine by thermogravimetry analysis (TG, red line), TG derivative in 1st order (DTG, pink line) and differential thermal analysis (DTA, black line).

The thermal behavior of the majority of pharmaceutical ingredients in gel formulations is illustrated in Fig. 2. The temperature range for each sample (x-axis) was adjusted to better visualize the phenomena. A mass loss of 7.3% was observed for [Carbopol 940], corresponding to the dehydration of the sample (TG curve, red line). At 138.8 °C (T_onset_), there is a small endothermic signal (ΔH 10.7 J g^−1^, DTA curve, black line), without mass loss (Δm 0), which corresponds to the “thermal history” of the material and is typical of polymers^57^. Carpobol had a thermal stability up to 180 °C with decomposition in four stages (TG curve, red line), as confirmed by DTG (pink line), Δm 81.5%. A broad endotherm of volatilization was observed for 70% ethanol in the DTA curve (black line), which is a phenomenon that requires 1.84 kJ g^−1^ and Δm 97.5%. A 6.7% gradual mass loss (TG curve, red line) was observed for glycerol, with a corresponding heat absorption (DTA curve, black line). Decomposition occurred in a single step at ~ 150 °C (TG/DTG, red and pink lines, respectively). A thermal stability up to ~ 152 °C was observed for triethanolamine, with a total loss of mass at temperatures up to ~ 300 °C (TG curve, red line).

**Figure 3:**
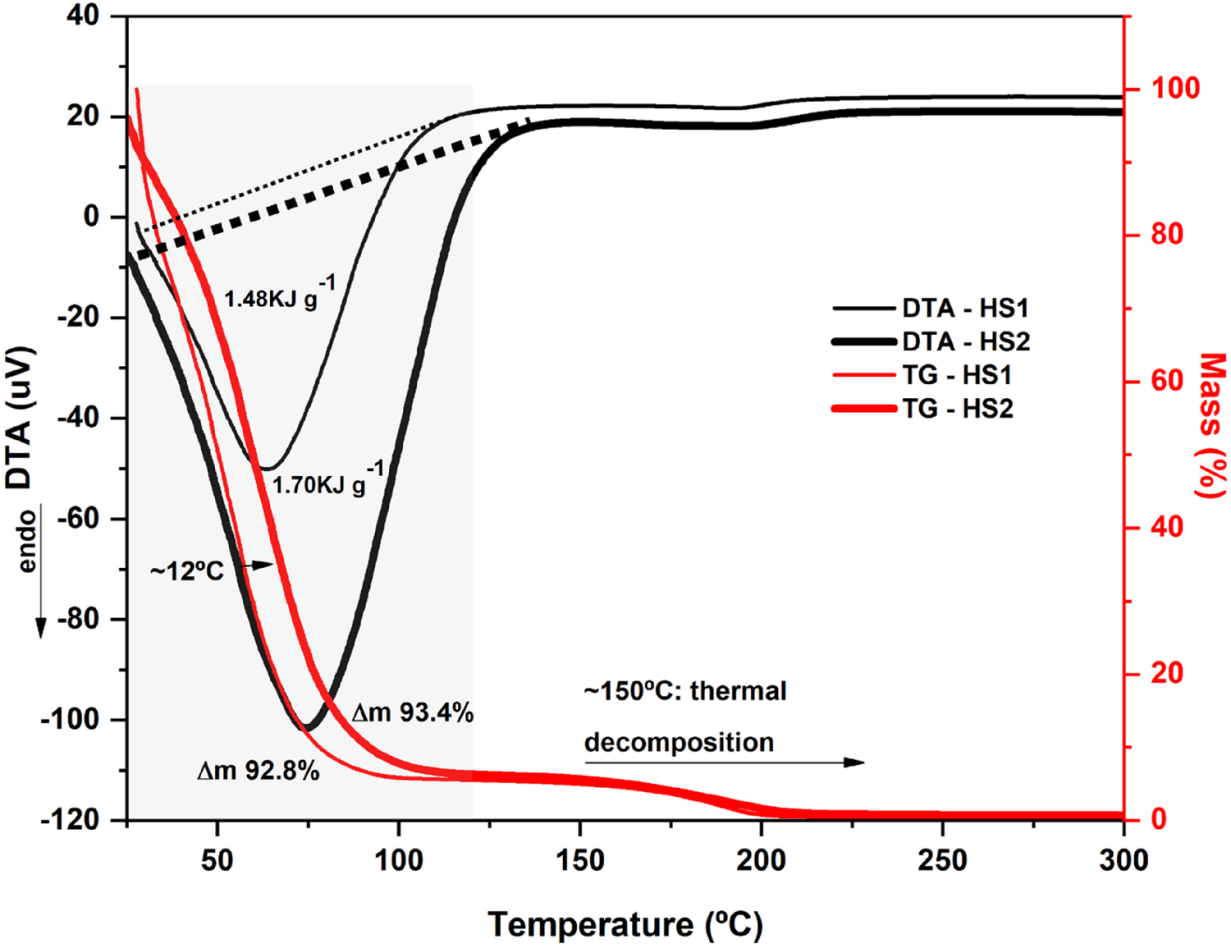
Thermal properties of gel alcohol in the hand sanitizer without (HS1—thin line) and with (HS2—thick line) glycerol, by thermogravimetry (red line) and differential thermal analysis (black line).

The thermal behavior of the gels is presented in Fig. 3. In the first stages of heating (30–120 °C), a broad volatilization endotherm of the hydroalcoholic vehicle is observed in both HS1 and HS2, as expected. DTA curves (black lines, thin for HS1 and thick for HS2, shaded region of Fig. 3), confirmed this phenomenon. HS1 required 1.48 kJ g^−1^ for volatilization of the hydroalcoholic vehicle. This value was calculated by integrating the curve (dotted line) in TAdata software Shimadzu, with Δm 92.8% (TG curve, thin red line). HS2 required 1.70 kJ g^−1^. This slightly higher value resulted from the presence of glycerol, which contributed to polar interactions with the polymer base, reduced the vapor pressure of the gel and delayed the occurrence of this phenomenon by 12 °C, as observed in the temperature range (x axis). For HS2 this phenomenon exhibited Δm 93.4% (TG curve, thick red line). Both gels decomposed above 150 °C.

## Conclusions

Refined crude glycerol obtained from biodiesel production was introduced as a humectant in the hand sanitizer formulation, and a simple, rapid method for quantifying ethanol in ethanol-based gel hand sanitizers by HS-GC/FID was employed. Finally, even after the COVID-19 pandemic, the demand for ethanol-based hand sanitizers remains high, and the method described was shown to furnish excellent results regarding the effectiveness as a hand sanitizer. It also represents an alternative use for the glycerol being produced on a large scale by biodiesel processes. The product satisfies the requirements of WHO that preconize formulation containing 1.45% glycerol as an humectant to protect HCWs skin against dryness and dermatitis^34^.

### Supplementary Information

Supplementary Information.

## Data Availability

In this study, data availability statements or the datasets generated and analyzed during the current work are available from the corresponding author on reasonable request.
