# Supplementary material for: Purification and use of crude green glycerol from the transesterification of triglycerides in the formulation of an alcohol gel hand sanitizer
Source: Sci Rep. 2024 Mar 6;14:5510. doi: 10.1038/s41598-023-49422-5 (PMC10917745; doi:10.1038/s41598-023-49422-5)
Supplement: Supplementary file 1 — Supplementary Information. [file 41598_2023_49422_MOESM1_ESM.docx]

**Purification and use of crude Green Glycerol from the transesterification of triglycerides in the formulation of an alcohol gel hand sanitizer**

Tassio Trindade Mazala^1^, Mateus Costa Viana^1^, Guilherme Carneiro^1^, David Lee Nelson^1^, Maria B. de Freitas-Marques^2,4^, Bruno Spinosa De Martinis^3^, Jacques Florêncio^3^, Fernanda Marur Mazzé^5^, Severino G. Domingos da Silva^5^, Sandro L. Barbosa^1*^

^1^Department of Pharmacy, Universidade Federal dos Vales do Jequitinhonha e Mucuri-UFVJM, Campus JK, Rodovia MGT 367 - Km 583, nº 5.000, Alto da Jacuba, CEP 39100-000, Diamantina/MG, Brazil. e-mail: [tassio.mazala@ufvjm.edu.br](mailto:tassio.mazala@ufvjm.edu.br) (TTM); mateus.viana@ufvjm.edu.br (M.C.V.); [guilherme.carneiro@ufvjm.edu.br](mailto:guilherme.carneiro@ufvjm.edu.br) (G.C.); [dleenelson@gmail.com](mailto:dleenelson@gmail.com) (D.L.N.); [sandro.barbosa@ufvjm.edu](mailto:sandro.barbosa@ufvjm.edu) (S.L.B.).

^2^Department of Chemistry, Instituto de Ciências Exatas, Universidade Federal de Minas Gerais. Av. Antônio Carlos, 6627, Pampulha, 31270-901, Belo Horizonte - Minas Gerais - Brazil. e-mail: [betanialf@hotmail.com](mailto:betanialf@hotmail.com) (M.B.F.M.)

^3^Universidade de São Paulo, Faculdade de Filosofia, Ciências e Letras de Ribeirão Preto, Av. Bandeirantes, 3900, Ribeirão Preto, SP 14040-900, Brazil. e-mail: martinis@usp.br (B.S.M); jacquesflorencio@usp.br (J.F)

^4^Curso de Farmácia. Faculdade de Minas, Faminas-BH. Av. Cristiano Machado, 12001, Vila Clóris, 31744-007, Belo Horizonte - Minas Gerais - Brazil.

^5^Institute of Chemistry, Federal University of Rio Grande do Norte, Natal, RN 59072-970, Brazil. e-mail: [fernanda.mazze@ufrn.br](mailto:fernanda.mazze@ufrn.br) (F.M.M); [georgemsm@gmail.com](mailto:georgemsm@gmail.com) (S.G.D.S)

*Corresponding author. Tel.: +55-38-35321234; fax: +55-38-35321234; e-mail: [sandro.barbosa@ufvjm.edu.br](mailto:sandro.barbosa@ufvjm.edu.br)

_____________________________________________________________________________________

**Abstract**: Purpose: The aim of this study was to produce an alcohol gel hand sanitizer containing green glycerol.

Methods: Crude glycerol was purified using chemical and physical treatments, The sanitizer was prepared using 71.100 g of 99.3º GL ethanol, 28.0 g H2O, 0.5 g of Carbopol 940®, 5 drops of triethanolamine (pH 5-7), and glycerol (1.5% w/w). The thermal behavior of the ethanol, carbopol, triethanolamine, glycerol, and alcohol gels were evaluated using Thermogravimetry and Differential Thermal Analysis. The apparent viscosity was obtained using a rotary viscometer. The determination of in vitro spreadability was achieved by an adaptation of the Knorst method. The ethanol content was measured by headspace gas chromatography using a flame ionization detector.

Results: The thermal behavior of the gels was influenced by the presence of glycerol, which confirms the possible network interactions formed. The relative densities of the samples were between 0.887-0.890 g/cm³. No alteration of the pH of the formulation resulted from the incorporation of glycerol. The apparent viscosities of the alcohol gels were greater than 20,000 cP. No alteration in the in vitro spreadability of the gel alcohol (530.6 mm²) resulted from the addition of glycerol.

Conclusions: Hand sanitizer was produced using glycerol from a transesterification reaction. It was highly effective and exhibited improved dermatological characteristics. It represents an alternative use for the glycerol being produced by biodiesel processes. The product satisfied the requirements of WHO that preconize a formulation containing 1.45% glycerol as an emollient to protect skin against dryness and dermatitis.

*Keywords:* hand sanitizer; glycerol preparation; triacylglycerides; transesterification; quantification of ethanol; glycerin alcohol gel.

_____________________________________________________________________________________

**Characterization of the gel alcohol hand sanitizer**

Characterization of gel alcohol in the hand sanitizer with (HS2) and without (HS1) glycerol

| **Parameters** | **HS1** | **HS2** |
| --- | --- | --- |
| pH | 7.54 ± 0.13 | 7.69 ± 0.05 |
| Relative density (g/cm³) | 0.890 ± 0.006 | 0.887 ± 0.0009 |
| Apparent viscosity (cP) | 27070 ± 62 | 27040 ± 35 |
| *In Vitro* spreadability (mm²) | 530.6 ± 0.82 | 530.6 ± 0.82 |

**Ethanol Concentration**

Ethanol concentration determined in the samples (HS 1 and HS 2)

|  | Ethanol concentration  (%) |
| --- | --- |
| Sample HS 1 | 79.3 |
| Sample HS 2 | 87.4 |
